# Liquid Metal Composites‐Enabled Real‐Time Hand Gesture Recognizer with Superior Recognition Speed and Accuracy

**DOI:** 10.1002/advs.202305251

**Published:** 2024-01-26

**Authors:** Yi Chen, Zhe Tao, Ruizhe Chang, Yudong Cao, Guolin Yun, Weihua Li, Shiwu Zhang, Shuaishuai Sun

**Affiliations:** ^1^ CAS Key Laboratory of Mechanical Behavior and Design of Materials School of Engineering Science University of Science and Technology of China Hefei Anhui 230026 China; ^2^ Cambridge Graphene Centre University of Cambridge Cambridge CB3 0FA UK; ^3^ Faculty of Engineering and Information Sciences University of Wollongong NSW 2522 Australia

**Keywords:** LMMRE, master‐slave control, motion intention recognition, pressure sensing

## Abstract

Prosthetic hands play a vital role in restoring forearm functionality for patients who have suffered hand loss or deformity. The hand gesture intention recognition system serves as a critical component within the prosthetic hand system. However, accurately and swiftly identifying hand gesture intentions remains a challenge in existing approaches. Here, a real‐time motion intention recognition system utilizing liquid metal composite sensor bracelets is proposed. The sensor bracelet detects pressure signals generated by forearm muscle movements to recognize hand gesture intent. Leveraging the remarkable pressure sensitivity of liquid metal composites and the efficient classifier based on the optimized recognition algorithm, this system achieves an average offline and real‐time recognition accuracy of 98.2% and 92.04%, respectively, with an average recognition speed of 0.364 s. Thus, this wearable system shows advantages in superior recognition speed and accuracy. Furthermore, this system finds applications in master‐slave control of prosthetic hands in unmanned scenarios, such as electrically powered operations, space exploration, and telemedicine. The proposed system promises significant advances in next‐generation intent‐controlled prosthetic hands and robots.

## Graphical abstract

This work proposes a real‐time motion intention recognition system utilizing liquid metal composite sensor bracelets. The sensor bracelet detects pressure signals generated by forearm muscle movements and combines them with pattern recognition to recognize hand gesture intent in real‐time with superior recognition speed and accuracy.

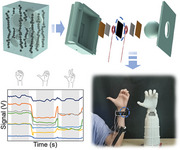

## 1 Introduction

Robots with real‐time controllable prosthetic hands have been well developed in advanced industries in recent years.^[^ ^1^ ^]^ Most prosthetic hands consist of many joints to mimic the dexterity of the human hand. However, the intention recognition in prosthetic hand users is still understudied, which prevents the prosthetic hands from fully utilizing their capabilities. Recently, the myoelectric signal (electromyography, EMG) is most commonly used to recognize motion intention.^[^ ^2^, ^3^, ^4^, ^5^, ^6^, ^7^ ^]^ In addition, the electroencephalogram (EEG),^[^ ^8^, ^9^ ^]^ B‐mode ultrasound,^[^ ^10^, ^11^, ^12^ ^]^ and visual technology^[^ ^13^, ^14^, ^15^ ^]^ are also widely used.

The motion intention of the user can be revealed by translating the EMG patterns generated by different muscle activities. However, some notable shortcomings limit the application of this method.^[^ ^16^, ^17^, ^18^, ^19^, ^20^, ^21^ ^]^ For example, the signal interferences caused by electromagnetic radiation, sweat, and mechanical stress often lead to low recognition accuracy and short working duration. In addition, the EEG signals are challenging to process because they undergo complex changes as they traverse the meninges, skull, fascia, and scalp.^[^ ^22^, ^23^, ^24^ ^]^ Ultrasound signals are presented as images, which are difficult to process and have poor real‐time performance.^[^ ^10^, ^25^, ^26^, ^27^ ^]^ The visual signal is collected by the high‐speed camera with complex equipment systems, thus showing limited application scenarios.^[^ ^28^ ^]^

Compared with them, the force sensor‐based limb motion intention recognition system shows the advantages of high signal stability, simple signal processing, small sensor size, and wearability, which address the issues of the above techniques.^[^ ^29^, ^30^, ^31^, ^32^ ^]^ However, its recognition accuracy depends on the sensitivity and stability of the force sensor. In our previous work, we created a liquid metal composite (Liquid Metal‐filled Magnetorheological Elastomer, LMMRE) with anisotropic and unconventional piezoconductivity. More importantly, LMMRE has the advantages of high sensitivity, great flexibility, and good processability, making it suitable for use as a force sensor.^[^ ^33^, ^34^, ^35^, ^36^ ^]^

In this work, we develop and demonstrate a real‐time limb motion intention system based on the liquid metal composites‐enabled force sensing bracelet. The intention recognition involves the analysis of complex data. To solve this problem, we use machine learning technology to improve the recognition accuracy.^[^ ^37^, ^38^, ^39^, ^40^, ^41^ ^]^ A robust statistical classification algorithm, the Support Vector Machine (SVM), is employed to identify the motions. We demonstrate the excellent gesture recognition performance of this system on a master‐slave control prosthetic hand.^[^ ^42^, ^43^, ^44^, ^45^ ^]^ This system has the advantages of fast response, high recognition accuracy, good wearability, and wide scenario versatility. Moreover, this system can recognize gestures without equipment on the hands, and can therefore be used for gesture intention recognition and prosthetic control for people with hand disabilities.^[^ ^46^ ^]^

## 2 Experimental Section

During the limb motion, the forearm muscles drive the fingers to produce different gestures. Through the LMMRE sensor, this system can convert the acquired forearm muscle tension signals into piezoresistive signals. The system adopts pattern recognition technology to perform real‐time recognition of hand gestures through model training and recognition. The following sections detail the preparation of the LMMRE, establishment of the sensor bracelets, and gesture recognition algorithm.

### 2.1 Preparation of the LMMRE

**Figure 1:**
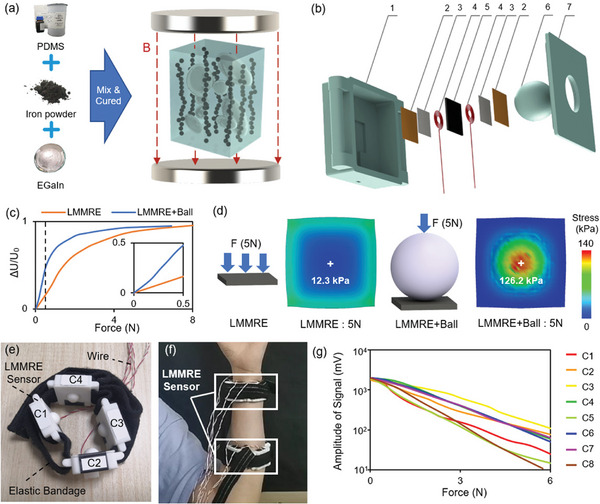
LMMRE sensor production and performance. a) Production of the LMMRE. b) Structure of the LMMRE sensor (1. box, 2. insulation layer, 3. conductive silver tape, 4. electrode, 5. LMMRE, 6. plastic ball, 7. cover). c) Voltage – pressure curve of LMMRE sensor with/without plastic ball. d) Simulation of stress distribution in LMMRE sensors with/without a plastic ball. e) Physical picture of LMMRE sensor bracelet. f) Wearing a picture of the LMMRE sensor bracelet. g) Voltage–pressure curve for 8 LMMRE sensing units.

The LMMRE is a pressure‐sensitive piezoresistive material developed in our previous work, which exhibits anisotropic conductivity and a piezoresistive effect.^[^ ^47^ ^]^ **Figure** **1**a shows its fabrication method. This composite consists of three raw materials: polydimethylsiloxane (PDMS, PDMS base/curing agent mass ratio of 9:1), carbonyl iron microparticles (2‐5 µm), and liquid metal (EGaIn, Eutectic Gallium‐Indium). The raw material mass ratio of LMMRE is PDMS: Fe microparticles: EGaIn of 1:4:1 (volume ratio of 1:0.495:0.154). The EGaIn liquid metal was purchased from Sigma‐Aldrich (Cat no: 495 425), Australia. The SYLGARD 184 Silicone Elastomer Curing Agent and SYLGARD 184 Silicone Elastomer Base were purchased from Dow Corning, USA. The carbonyl iron was purchased from Sigma–Aldrich (Cat no: 44 890), USA.

The ingredients were put sequentially into a clear plastic cup and then mixed by a high‐speed electric stirrer (400 rpm) for 2 min. The electric stirrer was equipped with a plastic mixing stick with a diameter of 4 mm. During this process EGaIn was broken into droplets with a diameter of 6–25 µm. Then the mixture was degassed in a vacuum chamber to remove air bubbles. Finally, we poured the mixture into an iron mold (100 × 100 × 1 mm) and placed the mold in a uniform magnetic field with a magnetic field strength of 1 Tesla (the magnetic field direction was perpendicular to the mold). The mixture was cured at 80 °C for 20 min to obtain anisotropic LMMRE composites.

### 2.2 LMMRE Sensor Bracelet

Harnessing the high sensitivity of LMMRE, a pressure‐sensing bracelet based on it was developed. Figure 1b shows the LMMRE sensing unit consisting of LMMRE (10 × 10 × 1 mm), electrode, conductive silver tape, insulation layer, sensor box, and a solid plastic ball. The 6 mm diameter electrodes are wound from 0.2 mm diameter conductive Teflon wire(purchased from Shenzhen Chengxing electronic wire direct sales department, made of conductive copper wire wrapped around Teflon insulation). To fabricate the core of the LMMRE sensing unit, we place the electrodes on both sides of the LMMRE. Conductive silver tape provides a tight contact between the electrode and the LMMRE. Then, the LMMRE, the electrodes, and the conductive silver tape with insulation tape are encapsulated. The size of the LMMRE sensing unit shell is 42 × 30 × 10 mm, the size of the cover plate is 30 × 30 × 2 mm, and the diameter of the plastic ball is 12 mm. And there is 11 mm diameter hole in the center of the cover, which serves as a limiting hole for the plastic ball so that the plastic ball will not be shifted. These parts are light‐curing 3D printed. The core of the LMMRE sensing unit is fixed inside the shell box together with the plastic ball. The sensing box enhances sensor sensitivity, secures the sensor, protects the sensor structure, and enhances the stability of the response. The wires (Teflon wire with a diameter of 0.2 mm) are led from the side of the box. We combined four LMMRE sensing units into a sensing bracelet using an elastic bandage (width of 20 mm). The LMMRE sensing unit is secured to the forearm using elastic straps to prevent its movement.

The solid plastic ball in the above sensing bracelet structure has much higher stiffness than the LMMRE material and is a sphere structure with point contact with both the forearm surface muscles and the LMMRE sensing unit. The plastic ball can concentrate the stress generated by the tensing forearm muscle to the LMMRE sensing unit, which enhances the signal acquisition effect. By connecting the LMMRE sensing unit in series with a 1 MΩ resistor and applying a voltage of 5 V, its divided voltage signal can be obtained. The force sensitivity of the LMMRE sensing unit calculated based on this divided voltage signal can be written as

(1) $$sensitivity\;=\frac{1}{U_{0}}\;\;\frac{\Delta U}{\Delta F}=\frac{U-U_{0}}{U_{0}\times \left(F-F_{0}\right)}\;$$

where *U* and *F* are the divided voltage and force on the LMMRE sensing unit respectively, U_0_ and F_0_ are the voltage and force at the previous moment. Figure 1c shows an order of magnitude increase in pressure sensitivity when the LMMRE sensor is mated with a plastic ball. At 0.5 N, the sensitivity (0.456/N) is 2.7 times that without the plastic ball (0.165/N). To explain this phenomenon, we performed finite element simulations for both structures. Figure 1d shows the stress simulation of the LMMRE with and without the plastic ball. Without the plastic ball, the LMMRE sheet is uniformly stressed. Under a force of 5 N, the stress on the central point of LMMRE is only 12.3 kPa. In conjunction with the plastic ball, the pressure is concentrated on the center of the LMMRE sheet. According to the simulation results, the stress at the center of the LMMRE sheet can reach 126.2 kPa, which is 10 times that without the plastic ball. The plastic ball effectively amplifies small forces (muscle tension) through stress concentration, leading to more dramatic resistance changes of the LMMRE, thereby significantly improving the sensitivity of the sensing bracelet.

As shown in Figure 1e, an LMMRE sensing bracelet consists of 4 LMMRE sensing units fixed on elastic straps. By adjusting the elastic straps, the sensor can simultaneously measure muscle stress signals at any position on the arm. This system contains 2 LMMRE sensing bracelets to collect muscle tension signals. The bracelets are worn as shown in Figure 1f. The 8 LMMRE sensing units exhibit a stable and similar exponentially linear pressure response. Figure 1g shows that the voltage drops by 2 orders of magnitude as the pressure increases from 0 to 6 N, reflecting the high sensitivity of the LMMRE sensor. Figure 1g shows that the initial test performance of each LMMRE varies somewhat due to the fabrication process and other reasons. These differences can be eliminated in the subsequent system design and will not affect the gesture recognition results(see Text S1, Supporting Information, for details).

### 2.3 Real‐Time Gesture Recognition System

**Figure 2:**
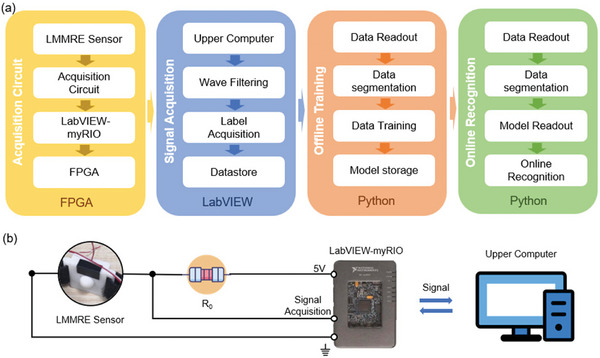
Structure of LMMRE online gesture recognition system. a) Data flow diagram of LMMRE online gesture recognition system. b) Architecture diagram of LMMRE online gesture recognition system.

According to **Figure** **2**a, the real‐time gesture recognition system in this experiment consists of four parts: acquisition circuit, signal acquisition system, model training program, and online recognition program. Figure 2b shows the acquisition circuitry based on the myRIO embedded system development platform (myRIO). The LMMRE sensing unit is connected in series with a 1 MΩ divider resistor. myRIO provides 5 V DC power and collects the voltage signal from the sensing unit, which is transmitted to the upper computer via the Field Programmable Gate Array (FPGA) module of myRIO. The signal acquisition in this system relies on the LabVIEW development environment (LabVIEW). The signals uploaded to the upper computer are filtered by smoothing in LabVIEW, and the smoothing window size is set to 15 data. After performing smoothing filtering, the small fluctuations of the signal are decreased and the interference of noise on the pattern recognition results is decreased. During the experiment, the signals are tagged with gesture labels by LabVIEW, and the signal data are stored locally on the computer.

The system is based on Pycharm Integrated Drive Electronics (IDE) and Anaconda IDE for model training and online recognition and utilizes a linear discriminant analysis classifier (LDA) and support vector machine classifier (SVM) for gesture recognition (see details in Figure S1, Supporting Information). The LDA classifier is a linear mapping of the input features into category scores; The SVM classifier is a generalized linear classification that binary classifies data in a supervised learning manner. The difference between them is the applicable scenarios. The LDA classifiers are suitable for scenarios with simple data structures and high requirements for classification speed. While the SVM classifiers are suitable for scenarios where the samples are not linearly separable.^[^ ^7^, ^48^, ^49^, ^50^, ^51^ ^]^ The offline training program includes data readout, data segmentation, data training, and model storage. The online recognition program includes data readout, data segmentation, model readout, and online recognition. The above system design can realize the functions of model training, model storage, and gesture recognition using the model stored in the local memory.

## 3 Experiment Results

### 3.1 Experiments

#### 3.1.1 Subjects

Four able‐bodied male subjects (aged 22–29, denoted as A1 to A4) and two able‐bodied female subjects (aged 22–28, denoted as A5 to A6) without a history of neuromuscular and joint diseases participated in the experiment. The impact of the physical condition of volunteers (including BMI) on test results is detailed in Table S1 (Supporting Information). Prior to the experiments, informed consent forms were obtained from all subjects.

#### 3.1.2 Wearing

**Figure 3:**
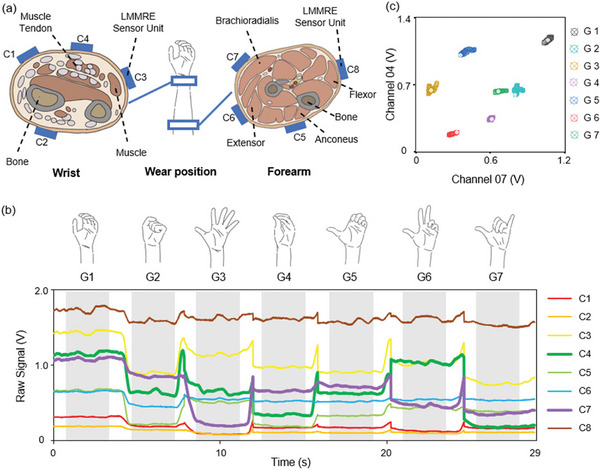
Experimental design. a) Schematic diagram of sensor placement (end and middle of forearm). b) Functional verification: dual‐channel sensing signals for gesture classification. c) The raw signals of the 8 sensors are compared with the gestures.

The LMMRE sensing units need to be placed within the firing range of the main few superficial muscles of the forearm (0.5 cm allowed deviation), so the position of each LMMRE sensor was chosen carefully, as shown in **Figure** **3**a. The 4 LMMRE sensing units are worn at the end of the forearm. The sensors placed on the inside (C4) and outside (C2) of the wrist are the main sensing signal acquisition units. The sensors placed on both sides of the wrist (C1 and C3) are the supplementary sensing signal acquisition units. Another 4 sensing units are worn on the mid‐forearm, all of which are the main sensing signal acquisition units. They are worn at the Anconeus (C5), Extensor (C6), Brachioradialis (C7), and Flexor (C8) muscles.

#### 3.1.3 Hand Gestures

To evaluate the performance of the Liquid Metal Composites‐enabled Sensor Bracelet for finger motion recognition, 7 gestures including rest state are chosen. As shown in Figure 3b, the 7 gestures are loose grip (G1), clenched fist (G2), five‐finger extension (G3), five‐finger grasp (G4), half grip (G5), thumb‐index‐middle finger extension (G6), thumb‐little finger extension (G7). The LMMRE sensing bracelet acquires real‐time signals, and when the gesture is changed, the signal value will be restored to a certain extent to that of the relaxation gesture (G1). After the gesture is completed, the signal value will be at a stable value.

#### 3.1.4 Experimental Program

During the offline experiment, the subjects were instructed to place their elbows on an armrest and lift their forearms with palms facing forward. All the 7 hand gestures mentioned above were repeated 3 times, each motion was held for 5 s, and only the data of the middle 3 s was analyzed. There was a 5 s rest between every two adjacent motions to avoid fatigue (the gesture corresponding to the rest time is loose grip (G1)).

During the real‐time experiment, the subjects were instructed to repeat the 7 gestures 3 times to collect training data. After the training, the subjects were instructed to repeat the 7 gestures for 1 time to test real‐time pattern recognition. Similarly, between every two adjacent motions, there was a 5 s rest (the gesture is loose grip (G1)).

#### 3.1.5 Functional Validation

Figure 3c shows the projection of the data on the two‐dimensional space. These data were collected from two of the most distinguishable signal channels. It is apparent that different motions can be clearly distinguished even with only two signal channels. Extending the signal channels to 8 increases the signal stability, improving the robustness of the system. This validates the effectiveness of the system for finger motion classification.

#### 3.1.6 Performance Metrics

The Classification Accuracy (CA) is used to evaluate the overall performance of the system. And CA is also used to represent the recognition accuracy of the system. CA is calculated as follows:

(2) $$CA\;=\frac{\mathrm{Number}\;\mathrm{of}\;\mathrm{correctly}\;\mathrm{recognized}\;\mathrm{motions}}{\mathrm{Total}\;\mathrm{number}\;\mathrm{of}\;\mathrm{testing}\;\mathrm{motions}}\;\times 100\%$$

To evaluate the real‐time performance of the system for finger motion recognition, two metrics applied in sEMG‐based HMI (human‐machine interface based on surface electromyography) were chosen.^[^ ^5^, ^27^, ^41^ ^]^ The definition of the two metrics was optimized according to the characteristics of this system. The two metrics are motion selection time (ST) and real‐time accuracy (RA). ST is the time interval between motion onset to the first right prediction of the targeted motion, which reflects the response speed of the system. RA is the recognition accuracy from the first correct prediction to the end of the action, reflecting the stability of the system(see details in Figure S2, Supporting Information).

### 3.2 Experiment Results

#### 3.2.1 Recognition Accuracy

**Figure 4:**
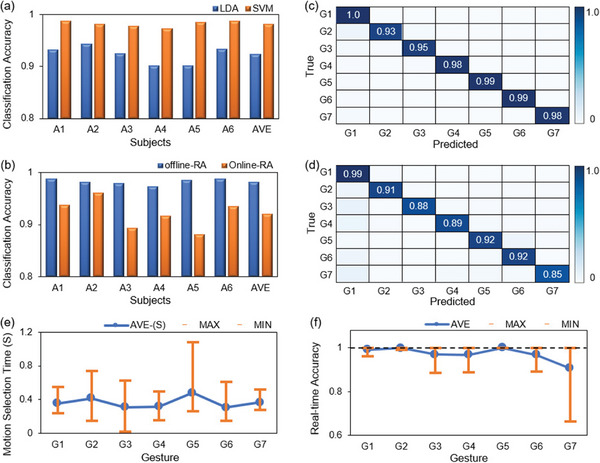
Experiment Results. a) Accuracy of offline recognition of gestures based on the LDA and SVM classifier for 6 subjects. b) Offline and real‐time gesture recognition accuracy for six subjects based on the SVM Aclassifier. c) Confusion matrix for offline gesture recognition. d) Confusion matrix for real‐time gesture recognition. e) Motion selection time (ST) for 7 gestures based on the SVM classifier. f) Real‐time accuracy (RA) of 7 gestures based on the SVM classifier.

**Figure** **4**a presents the average recognition accuracy of 7 gestures for each subject. The average recognition accuracy of the 6 able‐bodied subjects is 92.32% for the LDA classifier and 98.20% for the SVM classifier, respectively. In this system, the signals exhibit linear indistinguishability. Therefore, the recognition accuracy of the SVM classifier for each subject is higher than that of the LDA classifier. Thus, the SVM classifier was used for the motion intention recognition in the following experiments.

Figure 4b compares the difference between the offline and real‐time gesture recognition results based on the SVM classifier, where the offline average recognition accuracy is 98.2 ± 0.95% and the real‐time one is 92.04 ± 3.99%. In the real‐time recognition process, recognition errors may arise due to the rapid change of signals during the gesture transition. Another possible reason for the difference in accuracy between real‐time and offline is the different calculation methods. Offline methods usually use cross‐validation, which is close to the upper limit of the highest possible accuracy.^[^ ^4^, ^5^ ^]^

Figure 4c,d presents the confusion matrix of the offline and real‐time motion recognition results based on the SVM classifier. The data in the confusion matrix are the average recognition accuracy for each gesture, and the recognition data are from six volunteers. Their average recognition accuracy is ≈97.43% (offline) and 90.86% (real‐time) for each gesture motion, respectively. Due to the individual differences of the subjects, their G7 gestures were not uniform. This causes a slightly lower average recognition accuracy of G7 movements than that of other gestures.

#### 3.2.2 Online Evaluation Performance

Figure 4e,f shows the online recognition performance of the 6 subjects for the 7 gestures. In the figures, the blue line is the average value of the data and the red line is the range from the minimum to the maximum value for each type of data. As shown in Figure 4e, the average ST of the 7 gestures is 0.364s. Except for G2 and G5, the ST of the other 5 gestures is ≈0.3 s. It proves that this system can recognize motions quickly so that the user will not feel the delay in motion intention recognition. The G2 and G5 gestures show a longer gesture completion time (MC), which indirectly affects their ST data.

As shown in Figure 4f, the average RA of the 7 gestures is 97.21%, and the RA of each gesture is above 90%. The RA of the G7 gesture is lower than others due to the non‐standard G7 gestures of the subjects. Except for G7, the RA of the other 6 gestures was above 96%. It means that the system has a stable recognition effect during gesture holding process, which is crucial in the stable control of the prosthetic hand.

#### 3.2.3 Statistical Analysis

The data in Figure 4a are the average of the seven gesture recognition accuracies for each volunteer and the total average of the seven gesture recognition accuracies for all volunteers based on the LDA classifier and SVM classifier. The data in Figure 4b are based on the average offline and real‐time recognition accuracy of the 7 gestures of each volunteer. As well as the total average of all volunteers' 7 gestures offline and real‐time recognition accuracy. The values in Figure 4c,d are obtained from the average of six volunteers. The values in the error bars of Figure 4e,f are derived from the average of the experimental data from six volunteers.

## 4 System Application: Master‐Slave Control

From the above results, this real‐time motion intention recognition system shows high accuracy, fast response speed, and high stability. Another advantage of this system is that it detects gestures by monitoring forearm muscle activity rather than finger movements. In the case of hand dysfunction, simple gesture recognition can still be achieved through the tensor information of the superficial muscles in the forearm.^[^ ^30^, ^46^ ^]^Therefore, it can also be used for motion intention recognition and prosthetic hand control in people with hand dysfunction. The next experiment proves this by applying this system to the master‐slave control of a prosthetic hand (shown in Movie S1, Supporting Information).

**Figure 5:**
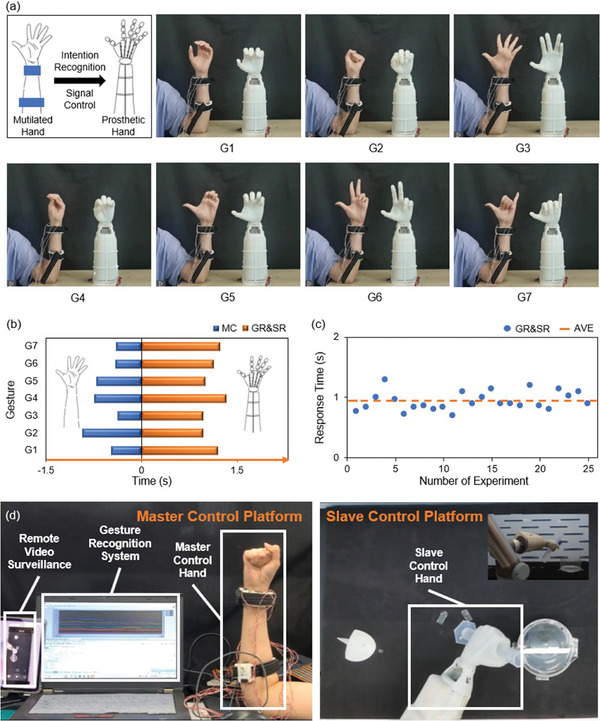
Master Slave Control. a) Photos of the experimental process using this system to control the manipulator. b) 7 gestures: time from the start to the completion of the movement (MC), time from the completion of the movement to the receipt of the control signal by the robot (gesture recognition time and signal reception time, GR&SR). c) G2 gestures: cycle test results of GR&SR. d) Remote operation platform for hazardous experiments.

This experiment collects signals through the LMMRE sensing bracelet and myRIO recognizes real‐time gestures through the SVM classifier and transmits the gesture signals to the prosthetic hand for gesture remote control through the serial port. In this experiment, seven hand gestures (G1–G7) are recognized in real‐time and transmitted to the prosthetic hand, as shown in **Figure** **5**a.

A practical prosthetic hand control system should have a 0‐error rate. However, as with this system, various current motion recognition methods are unable to achieve a 100% recognition rate. To solve this problem, this system sacrifices a certain recognition speed for a 0‐error rate. The average real‐time recognition accuracy of this system is 92.32%. On this basis, we take 150 recognized gesture signals to improve the recognition accuracy. This method filtered out the incorrectly recognized gesture signals, and its feasibility is verified by experiments.

In controlling the prosthetic hand, in addition to accurate recognition, fast recognition speed is required. Long recognition time will seriously affect the user's convenience and experience of operating the prosthetic hand. As shown in Figure 5b, the average motion completion time (MC, the time from motion start to motion completion) for the 7 gestures was ≈0.573 s. The average gesture recognition and signal reception time (GR&SR) for the 7 gestures was ≈1.118 s. In summary, it took an average of ≈1 s for gesture recognition and signal reception after the subject completed the gesture, which is within the acceptable range.

In addition, the system also shows good stability and reusability. We conducted five groups of repetitive experiments, and each group repeated five repetitions of the loose grip (G1) and clenched fist (G2). Figure 5c shows the GR&SR times for the five groups of G1. In the 25 repeatability experiments, the average GR&SR time of G1 was 0.939s with a standard deviation of 0.150 s, showing the high stability of this system.

Furthermore, our system enables master‐slave control of prosthetic hands, benefiting individuals engaged in hazardous tasks through remote operation. As demonstrated in Figure 5d and Movie S2 (Supporting Information), the system is used in a perilous chemical experiment involving sodium metal and water, known for its potential hazards due to the violent reaction and flammable gas production (hydrogen). Through the system in this work, the operator can safely control the prosthetic hand in real‐time from a distance, ensuring their safety while successfully completing the experiment.

In this experiment(shown in Movie S2, Supporting Information), the prosthetic hand serves as the Slave Control Hand, performing the actual experimental operation in the fume hood(acting as the Slave Control Platform). Wearing a gesture recognition bracelet on the forearm (the Master Control Hand), the operator remotely controls the prosthetic hand's movements from the gesture recognition platform (the Master Control Platform). The operator observes the Slave Control Platform via Remote Video Surveillance and sends real‐time gesture signals to the prosthetic hand through the gesture recognition system, guiding its operation.

The above experiments demonstrate the efficacy of the master‐slave control method based on the gesture recognition system developed in this work. It enables operators to conduct remote operations safely in hazardous or inaccessible environments, showing promising applications in fields of telemedicine, chemical industry, underwater operations, space exploration, and so on.

## 5 Conclusion

Based on LMMRE material with high‐pressure sensitivity, we design and develop the motion intention recognition system, comprising flexible sensing bracelets, a data acquisition system, a pattern recognition system, and serial transmission. The system recognizes finger motion through pressure changes on the forearm surface. The offline and online performance of this system for finger motion recognition has been evaluated in this work. Offline evaluation reveals that 98.2 ± 0.95% of hand motions in the test set are correctly predicted. In real‐time recognition, 92.04 ± 3.99% of the intended motions are completed with a selection time of 0.364s and an accuracy of 97.21%. Furthermore, when applied to prosthetic hand master‐slave control, the average time for gesture recognition plus signal transmission is 1.118 s. Notably, this system eliminates the need for hand‐mounted equipment to recognize gestures. It can therefore be used for motion intention recognition and prosthetic hand control for hand dysfunction groups. These outcomes validate the feasibility of the system in scenarios requiring dexterous finger motion recognition. Therefore, with its wide applicability across diverse user groups and usage scenarios, this system holds substantial potential for industry and healthcare applications.

## Conflict of Interest

The authors declare no conflict of interest.

## Supporting information

Supporting Information

Supplemental Movie 1

Supplemental Movie 2

## Data Availability

The data that support the findings of this study are available from the corresponding author upon reasonable request.
