# Supplementary material for: Liquid Metal Composites‐Enabled Real‐Time Hand Gesture Recognizer with Superior Recognition Speed and Accuracy
Source: Adv Sci (Weinh). 2024 Jan 26;11(37):2305251. doi: 10.1002/advs.202305251 (PMC11462307; doi:10.1002/advs.202305251)
Supplement: Supplementary file 1 — Supporting Information [file ADVS-11-2305251-s003.pdf]

ADVANCED SCIENCE
Open Access

Supporting Information

for *Adv. Sci.*, DOI 10.1002/advs.202305251

Liquid Metal Composites-Enabled Real-Time Hand Gesture Recognizer with Superior Recognition Speed and Accuracy

Yi Chen, Zhe Tao, Ruizhe Chang, Yudong Cao, Guolin Yun, Weihua Li, Shiwu Zhang and Shuaishuai Sun**

Supplementary Figure S1

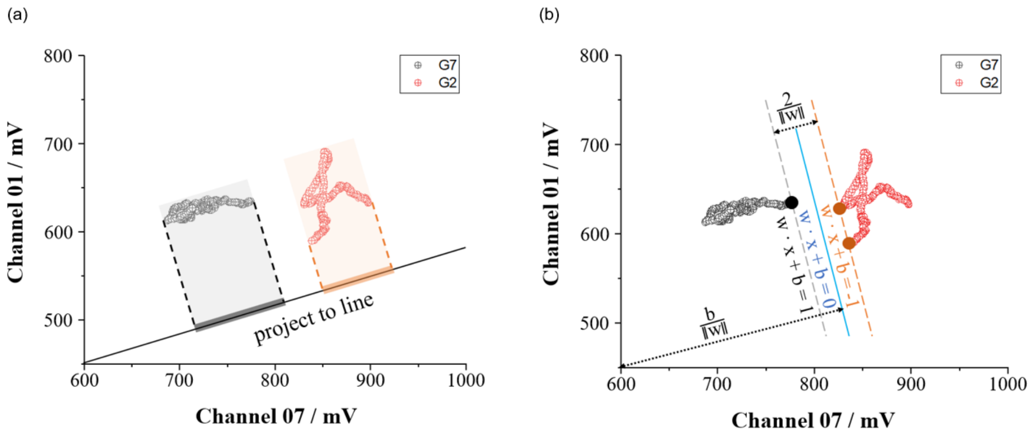

Figure S1: (a) Schematic diagram of linear discriminant analysis(LDA) classification principle in this system; (b)Schematic diagram of the Support Vector Machine(SVM) classification principle in this system

LDA classifier is a linear mapping that maps the input features into category scores[1]. Figure S1(a) shows the classification principle of some of the experimental data (G2 and G7 gesture data of the channel 01 and 07) in this paper in the LDA classifier.The LDA classifier projects the experimental data onto a certain straight line, linearizes the complex data, and finds the straight line that can categorize the various types of data as much as possible. The LDA classifiers are suitable for linearly differentiable simple signal scenarios..The LMMRE sensing bracelet consists of eight LMMRE sensing units, the data is more complex and has a lower fitness with the LDA classifier.

The SVM classifier is a generalized linear classification for binary classification of data by supervised learning[2,3]. Figure S1(b) shows the principle of classification in SVM classifier for some of the experimental data of this work. Some points where two categories are closest to each other become support vectors.The SVM classifier will find the support vectors of the data and find a straight line in order to make the separation vectors of the different categories of data as far away from each other as

possible.The SVM classifier solves the problem of classifying nonlinear data and avoids computational cost due to the excessive dimensionality of the data by utilizing the kernel function. Therefore SVM classifier is more suitable than LDA classifier for classifying nonlinear 8-channel data acquired from LMMRE sensing bracelet. The experimental results shown in the various types of experiments in the manuscript validate this conclusion.

Supplementary Figure S2

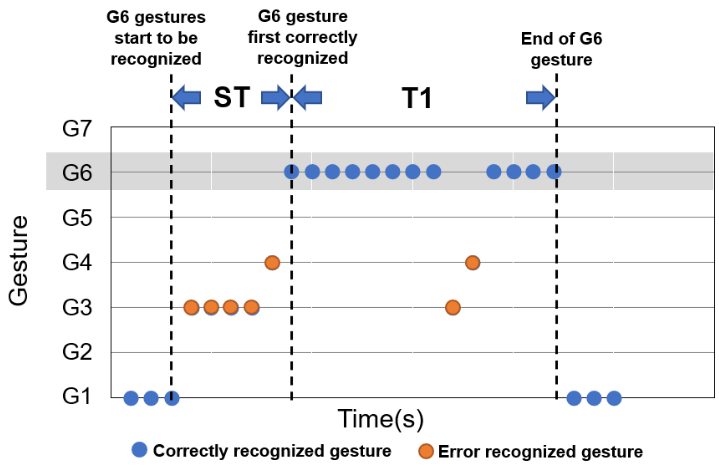

Figure S2: Real-time performance metrics illustration.

Figure S2 illustrates the definition and calculation of selection time (ST) and Real-time Accuracy (RA) by taking the recognition process of G6 gesture as an example.

Figure S2 shows ST is the time interval between motion onset to the first right prediction of the targeted motion, which reflects the response speed of the system. RA is the recognition accuracy from the first correct prediction to the end of the action(Percentage of the total number of correct gesture recognitions in time T1.), reflecting the stability of the system.

Figure 4(d)shows the confusion matrix for real-time gesture recognition. The confusion matrix is the specific value of the recognition accuracy of the seven gestures in the case of real-time gesture recognition. The recognition data of the confusion matrix is the overall data of a gesture from the beginning to the end of recognition, showing the overall recognition accuracy of the recognition effect, which can be used for the most direct judgment of the recognition effect of each gesture.

Figure 4(f) shows real-time accuracy (RA) of 7 gestures based on the SVM classifier. Real-time Accuracy (RA) is the recognition accuracy from the first correct prediction to the end of the action, reflecting the stability of the system. RA is a real-time performance metric that better reflects the real-time stability of the system than the confusion matrix.The overall trend of the data from RA and the more basic confusion matrix is consistent, and the gestures that are poorly recognized in the confusion matrix also have lower data in RA. The two sets of data characterize different meanings, but the overall trend is consistent also reflects the reliability of the experimental results.

Supplementary Movie S1

7Gesture-3X.mp4

Movie S1: Recognition of 7 Gestures to Control a Prosthetic Hand. Master-slave control of a prosthetic hand is realized by using the gesture recognition system of the paper. The specifics of the experiment are to recognize 7 gestures of the master control hand in real time and to operate the slave control hand. The video plays at 3x speed.

Supplementary Movie S2

Hazardous Tasks-3X.mp4

Movie S2: Master-Slave Control of Gesture Recognition Systems in Hazardous Tasks. This movie shows a chemistry lab technician performing a hazardous experiment with a flammable explosive by using the master-slave control of the manipulator with the system in the paper. This experiment fully demonstrates the effectiveness of the system in hazardous tasks. The video plays at 3x speed.

Supplementary Table S1

Num	Sex	Age	Height(cm)	Weight(kg)	BMI
A1	M	25	170	61	21.11
A2	M	23	170	73	25.26
A3	M	30	177	58	18.51
A4	M	23	178	80	25.25
A5	F	23	150	45	20.00
A6	F	27	168	52	18.42

Table S1: Volunteer physical condition data.

In the main text, Figure 4(a) presents the offline recognition accuracies based on the two classifiers are similar for all volunteers except for A4 volunteers, and the gesture recognition ability of the system is more stable. Table S1 demonstrates the diversity of the volunteer sample, two volunteers with a BMI of about 25 were searched for at the beginning of designing the experiment. Among them, A2 volunteers are fitness enthusiasts, and A4 volunteers exercise less, and the muscle content of their arms is less than other volunteers (including A2, a volunteer with the same BMI index of 25), which has negative impact on the present system based on pressure sensing for gesture recognition.

Furthermore, Figure 4(b)presents the difference recognition accuracies of the six volunteers from 2.1% to 10.42%, with the smallest gap between offline and real-time recognition accuracies for A2 volunteers and the largest gap between offline and real-time recognition accuracies for A5 volunteers. Same as the original reason in the offline gesture recognition experiment above, the A2 volunteer is a fitness enthusiast with a

thinner fat layer, which can control their muscles better and more stable than ordinary people, making the forearm surface muscle tension captured by each gesture more stable, and thus the recognition accuracy of real-time gestures is higher, and also the difference between the recognition accuracy and that of offline gestures is smaller. On the other hand, the A5 volunteer, a girl with a smaller body, had a slower speed of inter-gesture conversion in the experiment, and the interference data of inter-gesture conversion accounted for a larger proportion than that of the other experimenters, so the recognition accuracy of real-time gesture recognition was lower.

Supplementary Text S1

Figure 1 (g) shows that the voltage drops by 2 orders of magnitude as the pressure increases from 0 to 6 N, reflecting the high sensitivity of the LMMRE sensor. Figure 1(g) shows the different initial performance of each LMMRE sensor. Due to inevitable deviations in the preparation of LMMRE materials and the assembly of LMMRE sensing units, there are some differences between the force sensitivities of LMMRE sensing units. However, Figure 1(g) shows that the pressure response of each sensing unit is stable. Additionally, the 3.1.4 Experimental program of the manuscript shows each sensor is trained prior to gesture recognition. After each sensor is trained with data, signal acquisition and calibration are performed independently in subsequent experiments. Therefore it is only necessary that the LMMRE sensors have a stable response in use and are trained before use, and the initial differences have no effect on the data.

References

[1] Tharwat A, Gaber T, Ibrahim A, et al. Linear discriminant analysis: A detailed tutorial [J]. AI Commun, 2017, 30(2): 169-90.

[2] Oskoei M A, Hu H S. Support vector machine-based classification scheme for myoelectric control applied to upper limb [J]. IEEE Trans Biomed Eng, 2008, 55(8): 1956-65.

[3] Hsu C W, Lin C J. A comparison of methods for multiclass support vector machines [J]. IEEE Trans Neural Netw, 2002, 13(2): 415-25.
